# Supplementary material for: EnP1 exploits H2Aub-dependent epigenetic reprogramming to promote microsporidia proliferation in host cells
Source: PLoS Pathog. 2026 Jan 7;22(1):e1013853. doi: 10.1371/journal.ppat.1013853 (PMC12795459; doi:10.1371/journal.ppat.1013853)
Supplement: S1 Table — (DOCX) [file ppat.1013853.s005.docx]

**S1 Table.** Mass spectrometry-based profiling of EnP1 protein interactome

| **Description** | **Score** | **Sequence Coverage(%)** | **Peptides** |
| --- | --- | --- | --- |
| Histone H2B type 1-K | 549.39 | 69.05 | 15 |
| Histone H2A type 1-B/E | 488.92 | 46.15 | 9 |
| Putative histone H2B type 2-D | 73.92 | 28.66 | 7 |
| Isoform 3 of Nucleophosmin | 72.64 | 33.98 | 6 |
| Isoform 2 of Non-POU domain-containing octamer-binding protein | 65.33 | 38.48 | 15 |
| Isoform 2 of Dolichyl-diphosphooligosaccharide--protein glycosyltransferase subunit 2 | 60.01 | 29.59 | 11 |
| Isoform 2 of Glyceraldehyde-3-phosphate dehydrogenase | 58.52 | 55.29 | 10 |
| Isoform 2 of 5'-3' exoribonuclease 2 | 45.9 | 17.73 | 11 |
| Isoform G of Protein SON | 44.58 | 7.25 | 11 |
