# Supplementary material for: EnP1 exploits H2Aub-dependent epigenetic reprogramming to promote microsporidia proliferation in host cells
Source: PLoS Pathog. 2026 Jan 7;22(1):e1013853. doi: 10.1371/journal.ppat.1013853 (PMC12795459; doi:10.1371/journal.ppat.1013853)
Supplement: S2 Table — (DOCX) [file ppat.1013853.s006.docx]

**S2 Table.** List of primers for qPCR

| **Gene** | **Forward primer (5’-3’)** | **Reverse primer (5’-3’)** |
| --- | --- | --- |
| *SSU* (Eh) | TGACGGAAGGACACCA | CATCTCAGGGCATAACG |
| *BAP1* | CTGGCGAGCCCTTGAGTG | TGTGGGTCCTTCTCTGGTCA |
| *RING1A* | AGAATGCCAGCAAAACGTGG | CCAGCTTCTTTCGGCAGGTA |
| *RING1B* | GCAGACTGCATCATCACAGC | TACTAGGGCCTGCTTCCTGA |
| *USP16* | GGGGTTGTGACACCGAAAGA | TTCACCACCAAAGATGCGGT |
| *SLC7A11*(qPCR) | GTTGCTGGGCTGATTT | GAAGAGGCATGTGAAGG |
| *GAPDH* | GACCTGACCTGCCGTCTA | AGGAGTGGGTGTCGCTGT |
| *SLC7A11*(ChIP-qPCR) | TGTTGCTCACTACGAGTTGCT | AGCCTCCAGCATTACTCAGC |

| **Relative products** | **Resource** | **Identifier** |
| --- | --- | --- |
| **Antibodies** | | |
| Mouse anti-HA mAb | Abmart | Cat#M20003, RRID: AB_2864345 |
| Rabbit anti-HA mAb | Abclonal | Cat#AE105, RRID: AB_2943030 |
| Mouse anti-Flag mAb | Abways | Cat#AB0008, RRID: AB_2943672 |
| Rabbit anti-Flag mAb | Abclonal | Cat#AE092, RRID: AB_2940847 |
| Rabbit anti-SLC7A11/xCT mAb | Abclonal | Cat#A2413, RRID: AB_2863004 |
| Rabbit anti-RING1B/RNF2 mAb | Abclonal | Cat#A3564, RRID: AB_2863088 |
| Rabbit anti-H2AK9ac mAb | Beyotime | Cat#AF1207 |
| Rabbit anti-H2AK5ac mAb | PTM BIO | Cat#PTM-106RM |
| Rabbit anti-USP16 pAb | Abclonal | Cat#A5861, RRID:AB_2766611 |
| Rabbit anti-RING1A mAb | Abclonal | Cat#A19750 |
| Rabbit anti-H2Aub (K119) mAb | Cell Signaling | Cat#8240, RRID: AB_10891618 |
| Mouse anti-H3 mAb | Sino Biological | Cat#100005-MM01, RRID: AB_2860034 |
| Rabbit anti-H2A mAb | Abclonal | Cat#A3692, RRID: AB_2863118 |
| Rabbit anti-BAP1 pAb | Beyotime | Cat#AF6282 |
| Rabbit IgG | Beyotime | Cat#A7016, RRID: AB_2905533 |
| Alexa Fluor 488 Goat anti-mouse IgG (H+L) | Thermo Fisher | Cat#A32723, RRID: AB_2633275 |
| Alexa Fluor 594 Goat anti-rabbit IgG (H+L) | ZSGB‑BIO | Cat#ZF-0516, RRID: AB_2936330 |
| Mouse anti-GAPDH mAb | Servicebio | Cat#GB12002-100 |
| Mouse anti-Tubulin mAb | Abmart | Cat#M30109, RRID: AB_2916070 |
| Anti-mouse IgG-HRP | Proteintech | Cat#SA00001-1, RRID: AB_2722565 |
| Anti-Rabbit IgG-HRP | Proteintech | Cat#SA00001-2, RRID: AB_2722564 |
| **Critical commercial assays** | | |
| TIANamp Genomic DNA Kit | Qiagen | Cat#DP304 |
| RNA Easy Fast Cell kit | Qiagen | Cat#DP451 |
| RaPure DNA CleanUp Kit | Magen | Cat#MD010 |
| EndoFree Plasmid Mini Kit | Cwbio | Cat#CW2106S |
| Lipofectamine™ 3000 transfection reagent | Invitrogen | Cat#L3000001 |
| Nuclear Protein Extraction Kit | Solarbio | Cat#R0050 |
| KLD Enzyme Mix | New England Biolabs | Cat#M0554S |
| Seamless Cloning Kit | Beyotime | Cat#D7010 |
| HiScript III RT SuperMix | Vazyme | Cat#R323-01 |
| 2 × Phanta Max Master Mix | Vazyme | Cat#P515-01 |
| ChamQ SYBR qPCR Master Mix | Vazyme | Cat#Q311-02 |
| 2 × Taq Master Mix | Vazyme | Cat#P112-01 |
| Cell Counting kit-8 | Topscience | Cat#T005-00000005 |
| FastKing cDNA synthesis kit | Qiagen | Cat#KR116 |
| LDH Cytotoxicity Assay Kit | Beyotime | Cat#C0016 |
| GSH and GSSG Assay Kit | Beyotime | Cat#S0053 |
| Reactive Oxygen Species Assay Kit | Beyotime | Cat#S0033S |
| **Experimental models: Cell lines** | | |
| Human foreskin fibroblast cells | ATCC | Cat#CRL-2522, RRID: CVCL_3653 |
| Human embryonic kidney 293T cells | ATCC | Cat#CRL-11268, RRID: CVCL_1926 |
| EnP1 and GFP stable cell lines | This study | N/A |
| **Experimental models: Organisms/strains** | | |
| *Encephalitozoon hellem* | ATCC | Cat#50504 |
| **Oligonucleotides** | | |
| Primers for qPCR and ChIP-qPCR | This study | See Table S1 |
| si-BAP1 5’-CGUCCGUGAUUGAUGAUGAdTdT-3’ | This study | N/A |
| si-NC 5’-UUCUCCGAACGUGUCACGUdTdT-3’ | This study | N/A |
