# Supplementary material for: EnP1 exploits H2Aub-dependent epigenetic reprogramming to promote microsporidia proliferation in host cells
Source: PLoS Pathog. 2026 Jan 7;22(1):e1013853. doi: 10.1371/journal.ppat.1013853 (PMC12795459; doi:10.1371/journal.ppat.1013853)
Supplement: Attachment — Submitted filename: Response_to_reviewers-Final.docx [file ppat.1013853.s007.docx]

The point-by-point answers to the questions, comments, and suggestions are listed below.

***Reviewer Comments:***

***Reviewer #1***

***Comments:***

*Guan, et al describe the characterization of an interaction between EnP1 (from the intracellular microsporidian parasite, E. hellem) and H2A (from the host cell). This putative interaction was suggested from an EnP1-pulldown-proteomics experiment previously reported from the same group (ref 6). The authors confirm that EnP1 and H2A interact in pulldown assays, and show that EnP1 over-expression (or E. Hellem infection) leads to increased levels of ubiquitinated-H2A in host cells. Through a series of experiments, the authors piece together a possible pathway (illustrated in Fig 6), wherein: 1) EnP1 binds to H2A and down regulates BAP1 (not clear if EnP1 binding to H2A is related to its effect on BAP1?), 2) H2Aub accumulates on the promoter of the SLC7A11 gene, and 3) leads to increased production of SLC7A11, reduced ferroptosis, and greater parasite replication.*

*Overall, the data presented in each figure is generally clear and easy to interpret. While I have some questions about number and type of replicates throughout (technical vs biological), in general, I don't have major concerns about the execution of the experiments. I do, however, feel that there is something odd about the logic/argument throughout the manuscript, that gives me pause. In several places, the authors seem to transition into very focused set of experiments, without a clear chain of logic connecting them to the previous experiment/prior knowledge. I'm not sure if this is because their logic and/or additional experiments that helped to make the connection are just not presented; or this is because the experiments are not really connected, but have been spliced together to create a coherent story from a collection of experimental data. For example, see my comments on ln 147-160 and ln 161-164. The authors ultimately present a possible model for EnP1 function, but in the absence of a strong rationale behind the experiments, it is hard to assess whether is the most probable model, or simply one of many possible models (the others being left largely un-discussed and un-explored). For example, the model ultimately suggests the EnP1-induced changes in H2Aub leads to changes in SLC7A11 expression, and that is what favors parasite replication. But changing the levels of H2Aub seems like a pretty heavy-handed way for the parasite to regulate the expression of a single gene. Presumably, changing the levels of H2Aub would likely have pleiotropic effects throughout the transcriptome, but no other differentially expressed genes are discussed. I am concerned that the narrow focus of much of the text and experimentation may lead to erroneous conclusions, or at least a very incomplete picture of what is actually going on.*

**Response:**

We are deeply grateful to the reviewer for the thorough assessment and constructive feedback on our work. In response to the valuable comments, we have systematically addressed each concern through detailed point-by-point replies in our response letter. More importantly, a detailed exposition has been made concerning experimental data reproducibility and the robustness of research findings, serving to elucidate our original research intentions and optimize the investigation framework.

***Major Comments:***

*1. ln 120-122: An AlphaFold prediction is presented in Fig S1B as evidence supporting the interaction between EnP1 and H2A. I have a few concerns/suggestions here regarding rigor and reproducibility. First, there is no description in the methods of how this prediction was made. Which version of AlphaFold was used, was it run locally or on the web server, what parameters were used, and exactly what sequences were used (e.g., Uniprot IDs or some other traceable accession number)? Second, no metrics are presented establishing the predicted reliability of the model. The authors should minimally provide a figure with the model colored by pLDDT score, and a PAE plot, so reviewers and readers can assess the quality of the prediction. Third, since H2A normally assembles into an octamer with H2B, H3 and H4, it seems like one would want to predict EnP1 binding to the octamer instead of a monomer? Fourth, since these are not simply models downloaded from the AlphaFold database, they should be made available to readers either as a part of the supplementary materials, or through deposition in an appropriate database, such as* [*https://modelarchive.org/*](https://modelarchive.org/).

**Response:**

We thank the reviewer for their insightful comments regarding our structural predictions. In this study, we employed AlphaFold3 [1] via the publicly accessible web server (<https://alphafoldserver.com> ) to predict the interaction between EnP1 (accession: EHEL_010690) and H2A (UniProt ID: P04908). Given that H2A is a core component of nucleosomes, we performed predictions with the complete nucleosome octamer, which revealed potential interaction interfaces between EnP1 and the nucleosome (pTM=0.55, ipTM=0.53). Details of the methodology are provided in the Materials and Methods section of the revised manuscript (lines 712-727). The schematic diagram of the EnP1-nucleosome interaction indicates that EnP1 primarily binds to H2A and H2B within the nucleosome.

The predicted model of EnP1-nucleosome interaction: EnP1 (blue), H2A (red), H2B (green), H3 (yellow), H4 (purple):

While the overall complex model shows moderate confidence, we acknowledge the lower prediction reliability for EnP1 (pLDDT < 50) and consider these computational results as preliminary. These findings are in line with our experimental Co-IP validation. To help assess the model’s quality, we have included a prediction score plot of the EnP1-H2A complex in Figure S1D. Additionally, we conducted pull-down assays using EnP1-stable cell lines (Figure S1B), capturing binding targets (Figure 1A) and detecting H2A in the pull-down samples (Figure 1B). The H2A-EnP1 and full nucleosome-EnP1 complex models have been deposited in ModelArchive (<https://modelarchive.org> ), and are publicly accessible via the following link: <https://data.mendeley.com/datasets/bhscx3g5fc/1> . We have also included the pLDDT heatmap and PAE matrix for further model evaluation. We agree with the reviewer that further mechanistic studies using cryo-EM and other structural biology approaches are essential to fully characterize this interaction. This will be an important focus of our future work.

Figure S1B	Figure S1D

Figure 1A	Figure 1B

*2. ln 147-160: There is a bit of a conceptual leap here, or perhaps something that I don't understand. In my understanding, H2A and other histones can be modified in a number of different ways (Ub, methylation, acetylation...) and at many different sites (PMID: 35338361). In this section, the authors seem to jump from confirming the EnP1-H2A interaction, directly to linking K119-Ub of H2A to EnP1. How did they make this connection? Were other modifications tested/explored (and where is that data)? I think explaining the logic here will help the reader to understand, and I also think the chain of logic and experiments is important for scientific rigor. There needs to be a sounds reason for looking only at H2Aub, or there needs to be a more comprehensive analysis of histone modifications presented that explains why the authors have focused only on H2Aub.*

**Response:**

We previously reported that EnP1 can form a complex with H2B [2], and since H2A and H2B interact to form a heterodimer, which serves as a fundamental structural unit of the nucleosome, this suggested that H2A might also play a role in EnP1-mediated regulation. In fact, H2A was identified as the second most enriched protein after H2B in the EnP1 pull-down mass spectrometry data (Table S1), prompting us to explore whether H2A might also be involved in EnP1-mediated host regulation. Furthermore, we have established in previous work that EnP1 influence the monoubiquitination of H2B, and given the close functional relationship between H2Aub and H2Bub—both of which are involved in dynamic gene expression regulation and pathogen infection—we hypothesized that H2A may also play a synergistic or complementary role in these processes [3, 4]. This led us to focus on H2A monoubiquitination as a potential modification relevant to EnP1's activity.

H2A is indeed subject to various post-translational modifications, including acetylation, phosphorylation, and ubiquitination. In our study, we initially examined the acetylation levels at key lysine residues (K5 and K9) of H2A and observed no significant changes (Figure S2C-F), ruling out acetylation as a major factor in the context of EnP1 and infection. Given the well-established role of monoubiquitination in pathogen infection, as well as its known functional interplay with H2Bub in regulating gene expression, we decided to specifically examine H2Aub. This decision was based on the observation that both EnP1 expression and microsporidian infection significantly increased H2Aub levels. This finding provided a strong rationale for further investigate into the functional consequences and underlying mechanisms of this modification in the context of host-pathogen interaction.

In conclusion, while H2A can undergo several modifications, our focus on H2Aub was driven by the specific observation that EnP1 and infection conditions led to significant changes in H2Aub, as well as the established relationship between H2Aub and H2Bub in regulating gene expression during infection. This focus is grounded in the experimental data and the known role of monoubiquitination in pathogen regulation.

Figure S2C-F

*3. ln 161-164: Similar to my comment on ln 147-160, there is something I don't understand about why the authors focus only on one Ub ligase and one deubiquitinase. When I Google a little, I find papers mentioning more than 1 ligase (e.g., PMID: 39537923 mentions RING1A and RING1B) and at least one other DUB (PMID: 38918638). What am I missing? Why focus only on Ring 1B and BAP1? Might other ligases and deubiquitinases be involved? Were they tested and not shown?*

**Response:**

We appreciate the reviewer’s insightful comment. The focus on RING1B and BAP1 in our study stems from their well-characterized roles in the regulation of H2A monoubiquitination within the context of the PRC1 and PR-DUB complexes, respectively. Monoubiquitination of H2A is primarily regulated by the PRC1 complex, with RING1B serving as the catalytic subunit responsible for the ubiquitination of H2A, while RING1A acts as a regulatory component without intrinsic E3 ligase activity. Given this established functional division, we chose to focus specifically on RING1B as the E3 ligase responsible for H2A monoubiquitination [5, 6]. Conversely, the deubiquitination of H2A is predominantly mediated by the PR-DUB complex, with BAP1 acting as the core enzyme that plays the most critical role in this process [7, 8]. Other deubiquitinases, such as USP16 and USP21, exhibit limited specificity and minimal impact on H2A deubiquitination [9-11]. We have revised this paragraph for clarity (lines 209-233).

To directly address the reviewer’s concern, we tested the expression levels of both RING1A and USP16 at the transcriptional and protein levels. Our results, shown in Figure S2G-2R, revealed that neither EnP1 expression nor microsporidian infection significantly altered the protein levels of RING1A or USP16. These findings further support the conclusion that BAP1, rather than other ligases or deubiquitinases, is the primary regulatory enzyme responsible for the observed changes in H2Aub levels in our system.

Figures S2G-2R

*4. Fig 3B: This is a pretty subtle decrease in parasite levels, which may or may not be biologically meaningful. How does over-expression affect the host cells, and might that account for this difference? For example, is viability, doubling time, or metabolic activity affected? But setting that issue aside, some things about the methods were unclear to me and it would help to include additional information in the figure legend and methods. For example, in these experiments, where ~10^7 parasites were detected, how many spores were in the inoculum, and how many host cells were seeded in the dish? Were excess parasites washed away, and if so, how effective was this (as assessed by qPCR)? And for the data shown in the figure, are these technical replicates of the qPCR? Technical replicates for the infection? I ask these questions in part because the change in the figure is pretty small (less than 2 fold) but pretty significant (p<0.01), which I imagine would be difficult to observe with just a few biological replicates (for an experiment like this, in my opinion, biological replicates must be done on different days).*

**Response:**

We appreciate your feedback and would like to clarify a few points. All data presented in Figure 3B were derived from at least three independent biological replicates, with each replicate using parasites (Eh) from different batches, and infections were staggered by at least 24 hours to ensure biological robustness. Although the decrease in parasite levels appears subtle (less than twofold), this change is statistically significant (p < 0.01), and we believe it represents a biologically relevant inhibition of microsporidian proliferation, which we have thoroughly validated through multiple technical and biological approaches. Prior to these experiments, we confirmed that neither overexpression nor knockdown of BAP1 affected host cell viability compared to controls, eliminating potential confounding effects of differential cell survival on parasite replication (Figures S3D-E and Figures S3H-I). As for the inoculum, we utilized a consistent multiplicity of infection (MOI) of 10 for all experiments, and we explicitly include this detail in the Figure Legend (lines 304-316) and *Materials and Methods section* (lines 608-610) of the revised manuscript.

Figures S3D-E and Figures S3H-I

To accurately quantify the number of microsporidia infections, we employed an absolute quantitative qPCR method based on the microsporidia SSU gene. First, we infected cells at an MOI of 10, followed by three washes with PBS. Unwashed cells served as the control. Both the cells and their supernatants were collected, and total genomic DNA was extracted. The number of microsporidia in the samples was detected by qPCR to evaluate the washing efficiency. As shown in Figure S3A, the washing was effective. To further confirm the efficiency of washing under actual experimental conditions, we removed free and less viable microsporidia by washing with PBS at 12 hours post-infection. Genomic DNA was then extracted from both washed and unwashed cells, and the pathogen load was detected by qPCR. As demonstrated in Figure S3B, the washing efficiency was high, and the microsporidia counts were reasonable. This also confirms the sensitivity and feasibility of the method. Detailed protocols for microsporidian propagation, infection, and quantification have been comprehensively documented in the *Materials and Methods* section under “Assessment of Eh Proliferation” to enhance reproducibility and transparency (lines 602-618).

Figures S3A-B

*5. Fig 4E-4H: There isn't enough information in the figure legends and methods for me to understand these experiments. In particular, what is IgG meant to indicate? I think this is supposed to be "the control group" (ln 247), but what the control group doesn't seem to be described. Is this just polyclonal IgG? IgG against an irrelevant DNA binding protein?*

**Response:**

We thank the reviewer for their question regarding the experimental details of the ChIP assay. The IgG group uses non-specific immunoglobulin G (typically derived from the same species as the primary antibody, such as rabbit IgG, which is isologous to the H2Aub antibody we used) that does not recognize any target protein. Therefore, the DNA signal precipitated by IgG represents the background from such non-specific binding.

By comparing the signals between the target antibody group and the IgG group, false-positive results (such as non-specifically bound DNA fragments) can be eliminated. Simultaneously, this approach calibrates the background threshold. In data analysis (e.g., qPCR or high-throughput sequencing), the signal from the IgG group serves as the "background baseline." Only when the signal from the target antibody group is significantly higher than that of the IgG group (typically set at 2- to 3-fold or more) can it be considered a valid specific binding signal, thereby improving the accuracy of the results. This method also verifies the reliability of the experimental system. We have also provided additional clarifications in the figure legends (lines 370-371) and the *Materials and Methods* section (lines 768-769) of the revised manuscript.

*6. Throughout the manuscript, it is unclear how many replicates were done for each experiment, and whether those replicates are biological vs technical. My impression is that the error bars and spread of the data in many experiments may be too tight to be biological replicates in many cases, though I could be wrong. The most clear thing would be for the authors to state in each figure legend how many replicates were carried out, and in each figure legend (or in the methods) clearly describe what constitutes a replicate for each kind of experiment (e.g., 3 wells in the same dish, processed in parallel; 3 cultures grown and processed at least 1 day apart; 3 independently prepared parasite stocks infecting 3 different host cell cultures derived from separate vials thawed from freezer; etc).*

**Response:**

We have performed at least three independent biological replicates for each presented result. The cells or pathogens used in these three biological replicates were cultured and experimentally treated with intervals of at least 24 hours between each replicate. Additionally, the host cells used for infection were cultivated in separate batches. These details have been explicitly stated in the *Materials and Methods* section and each figure legend.

***Minor Comments:***

*ln 169-171: I suggest softening the conclusion here. The data presented is certainly consistent with this hypothesis, but I feel like there are still some gaps. BAP1 mRNA and protein levels seem to change in response to EnP1/infection, and H2Aub changes in the expected direction. But can it be said with certainty that the EnP1-induced change in BAP1 levels caused the change in H2Aub levels? It seems plausible to me that another unknown factor could be involved. Similarly, change in BAP1 levels are fairly modest. Is the change in BAP1 levels solely responsible? Might EnP1 also be modulating the activity of BAP1 post-translationally? As written, the authors rule out other plausible hypotheses without actually testing them.*

**Response:**

We have revised our conclusions to reflect a more cautious interpretation, as suggested. We agree with the reviewer’s point that while the data are consistent with the hypothesis, there are still gaps that warrant further consideration. The observed changes in BAP1 mRNA and protein levels in response to EnP1/infection, as well as the direction of H2Aub changes, support a potential relationship. However, it is not yet definitive that the EnP1-induced change in BAP1 levels directly caused the observed alteration in H2Aub levels. Regarding the mechanism underlying BAP1 downregulation, we hypothesize that it may be related to feedback regulatory effects triggered by the elevation of H2Aub (lines 230-233).

*Fig 3E: The abbreviation siNC doesn't appear to be defined, though it appears to be a control. While the sequence is given for the siRNA against BAP1, the sequence of siNC and its target don't appear to be specified.*

**Response**

The core function of siNC (non-targeting control siRNA) is to serve as a "non-specific targeting reference control," which helps distinguish the specific effects of target gene silencing from non-specific interference inherent in the experimental process. We have provided the sequence of siNC (si-NC 5’-UUCUCCGAACGUGUCACGUdTdT-3’) in the *key resources table* of Supporting Information file for full transparency and reproducibility.

*ln 233: Is SLC7A11 better described as a transporter? When I Google it, it comes up as a cystine/glutamate antiporter. It seems like its link to ferroptosis is pretty indirect.*

**Response:**

SLC7A11 is responsible for transporting extracellular cystine into cells [12]. Inside the cell, cystine is reduced to cysteine, which serves as an essential precursor for the synthesis of glutathione (GSH), the most critical intracellular antioxidant [13, 14]. Thus, the activity of SLC7A11 directly influences intracellular GSH levels and represents a central regulatory node in maintaining cellular antioxidant capacity and resistance to ferroptosis [15, 16].

*ln 240-241: I think here I think this should be referring to Fig 3A and 3B? Fig 4A and 4B Show BAP1 over expression and its effect on SLC7A11 (not on H2Aub). But then I don't think the text would refer to Fig 4A and 4B, so perhaps a sentence is missing, or this area needs a correction?*

**Response:**

We thank the reviewer for their careful evaluation. We have revised the description to clarify that BAP1 overexpression can alter the expression of SLC7A11 (lines 338-339).

*Fig 5J-5L: Why are full titration curves shown for 5E-5H, but not here?*

**Response:**

Figures 5E-H present full titration curves to investigate changes in the ferroptosis status of host cells treated with varying concentrations of the ferroptosis inducer Erastin, while BAP1 is either overexpressed or knocked down. These titration curves were essential to identify the specific Erastin concentrations (20 μM and 25 μM) that significantly promote or inhibit ferroptosis.

In contrast, Figures 5J-L focus on examining the dependence of EnP1's anti-ferroptotic effect on alterations in BAP1 expression levels. Since the ferroptosis in host cells was already confirmed to be induced at Erastin concentrations of 20 μM and 25 μM in Figures 5E-H, the full titration curves were not necessary for these experiments. Therefore, Figures 5J-L were conducted at these fixed Erastin concentrations to specifically assess the role of BAP1 expression in modulating EnP1’s effect on ferroptosis.

*ln 279-284: I am having a bit of trouble following this part. What does Fig 5I tell us that Fig 4B didn't already? I might be confused, but isn't the GFP line here as a frame of reference, to assess if BAP1OE has a different effect with and without EnP1 expression? So for each condition, don't we want to see 1) GFP alone, 2) GFP+BAP1OE, 3) EnP1 alone, 4) EnP1+BAP1OE?).*

**Response:**

We thank the reviewer for their valuable suggestion. We have now supplemented the experiment with the appropriate controls as recommended and performed three independent biological replicates to ensure the robustness and reproducibility of the results (Figure 5I and Figure S4).

Figures S5I	Figures S4

***Reviewer Comments:***

***Reviewer #2***

*In this manuscript, Guan et al. described the modulation of histone 2A (H2A) ubiquitination by Microsporidia infection. Elevated levels of ubiquitinated H2A (H2Aub) are induced by infection or overexpression of EnP1, a microsporidia effector that targets the host nucleus and has been previously implicated in epigenetic reprogramming. Moreover, the authors determine that SLC7A11 expression is upregulated due to enrichment with H2Aub at its promoter in EnP1 overexpression and infection conditions, resulting in resistance to ferroptosis inducers. The authors implicate diminished levels of BAP, a deubiquitinase known to act on H2Aub, as mediating the phenotypes dependent on elevated H2Aub. This work is valuable to the field as it provides mechanistic insight into microsporidia pathogenesis, which is generally poorly understood. Unfortunately, some significant problems/questions need to be addressed by the authors for publication.*

**Response:**

We sincerely thank the reviewer for their positive feedback and valuable suggestions. We have further refined the manuscript and supplemented additional data in the *Supporting Information*. Point-by-point responses to the specific issues raised by the reviewer are provided below. Relevant experimental data and discussions have been accordingly incorporated into the revised manuscript.

***Major Comments:***

*1. How is the total H2A accounted for in Figure 2? Is it possible that the same ratio of H2A is ubiquitinated when EnP1 is expressed or under infection conditions, as compared to control? What is the relevance of H3 in these blots? Are they a control for nucleosome formation? Are the levels of H3 used as a loading control, and if so, can H2A and H2Aub be normalized to them?*

**Response:**

The total H2A levels were measured in host cells under EnP1 overexpression and infection conditions, and we found that H2A levels remained unchanged (Figure S2A-2B). This indicates that any observed changes in H2A ubiquitination (H2Aub) are not due to alterations in total H2A expression, but rather reflect changes in the specific modification. While the ratio of H2Aub may vary between the EnP1-expressed or infected conditions compared to the control, this variation is primarily due to differences in EnP1 expression levels across conditions. Regarding H3, it is included as an internal reference for nuclear proteins and histone post-translational modifications, serving a role analogous to GAPDH for cytoplasmic proteins. In this context, H3 functions as a loading control to ensure equal amounts of nuclear proteins were analyzed. As shown in Figures 2B and 2D, the H2Aub band intensities were quantified after normalization to H3, thereby controlling for variations in sample loading and ensuring the reliability of the comparisons.

Figure S2A-B

*2. Does BAP bind to H2Aub when EnP1 is overexpressed or when cells are infected? Is BAP1 the only DUB for H2A? Additionally, why is only one E3 ligase tested?*

**Response:**

The deubiquitination of H2Aub (K119) is mediated by the PR-DUB complex, with BAP1 serving as the core enzyme that plays the most critical and dominant role [8, 17]. It binds to H2Aub and hydrolyzes the K119-linked ubiquitin chain. Both EnP1 overexpression and microsporidian infection interfere with this process, leading to reduced deubiquitination of H2A by BAP1. Although BAP1 is the most crucial deubiquitinase for H2A, it is not the only one; USP16 can also deubiquitinate H2A, albeit to a lesser extent. We examined changes in both transcriptional and protein expression levels of USP16 under EnP1 overexpression and microsporidian infection and found no alterations (Figure S2G-2L).

Monoubiquitination of H2A is catalyzed by the PRC1 complex, which consists of two subunits: RING1A and RING1B. RING1A primarily functions in a regulatory capacity, while RING1B carries out the ubiquitination modification. We also assessed the expression level of RING1A and found that neither EnP1 nor infection induced any changes, which is consistent with the results observed for RING1B (Figure S2M-2R).

Figures S2G-2R

*3.Can overexpression of Ring1 generate elevated levels of H2Aub? Does overexpression of Ring 1 result in the same infection phenotypes as siRNA against BAP? (Figure 3).*

**Response:**

Indeed, overexpression of RING1B exogenously elevates the level of H2Aub in host cells, but it functions in transcriptional repression [8, 18, 19]. In contrast, the increase in H2Aub resulting from BAP1 knockdown exerts a transcriptional activation effect on the key ferroptosis regulator SLC7A11, indicating opposing functional outcomes between the two manipulations—as mentioned and explained in the discussion section of the revised manuscript (lines 516-541). To simulate the physiological changes in host cells induced by EnP1 and to investigate the critical role played by altered SLC7A11 expression in this process, we employed BAP1 knockdown as an experimental approach. This approach better mirrors the infection-related phenotypes observed in our system.

*4. The experiments to generate the data in Figure 5I should be performed in triplicate and quantified.*

**Response:**

We have included additional biological replicates for Figure 5I. Furthermore, to ensure experimental rigor, we added a control group in which SLC7A11 expression was measured under BAP1 overexpression conditions to validate the effectiveness of the manipulation (Figure 5I and Figure S4).

Figures S5I	Figures S4

***Minor Comments:***

*In general, the authors over-rely on the reader having in-depth knowledge of their previous work on H2B and do not adequately explain the relevance or importance of that work to their current findings in this paper.*

*Frequently, the authors pose a question or hypothesis and then state an experimental conclusion without describing the experimental process used to derive the conclusion (lines 237-240 are an example of this). Additionally, when an experimental design is stated, it is often not described in sufficient detail to allow the reader to understand what is being quantified (lines 266 and 267 are examples of this). This makes the paper very hard to read.*

*In graphed data, individual biological replicates are not shown. This would help the reader determine whether the data is accurately represented by the blots or gels in the figures.*

*The image quality is very poor, making it difficult to read the text, especially in Figure 5.*

**Response:**

We thank the reviewer for their constructive feedback. In response, we have expanded the relevant sections of the manuscript by including additional details regarding the experimental design. Information about biological replicates has now been clearly stated in both the figure legend and the *Materials and Methods* section. Additionally, we have uploaded higher quality images for all figures, to ensure the text and data are legible. To further assist the reviewer, we have also made the original, high-resolution images available for download, which should allow for a better view of the data as originally submitted.

*Please explain how H2A acts as a chaperone for H2B (lines 89-90).*

**Response:**

The phrase "H2A acts as a chaperone for H2B" may have caused some confusion. In this context, H2A does not function as a "chaperone" in the traditional sense of assisting H2B in folding or refolding. Rather, H2A serves as a structural partner to H2B, with the two histones forming a heterodimer. This heterodimer is crucial for nucleosome assembly and the overall organization of chromatin. The interaction between these two histones is vital for maintaining chromatin structure and function, which, in turn, impacts various cellular processes, including the regulation of ferroptosis during pathogen infection. As a result, the term "chaperone" has been changed to "structural partner" in the revised manuscript (line 97).

*Please describe the inducible system used in Figure 1B and why it is necessary (lines 137-138).*

**Response:**

We have provided a description of the pRSFDuet vector and its usage in both the figure legend (Figure 1) (lines 168-169) and the *Materials and Methods* section (lines 690-692). As the pRSFDuet-1 vector contains an RSF 1030 origin and dual promoters, allowing the simultaneous expression of two genes. This makes it highly efficient for co-expressing two proteins (EnP1 and H2A in this paper) and is ideal for studying protein-protein interactions and complex biological pathways.

*Please state the residue on H2A that is ubiquitinated and identified by the antibody used in Figure 2 in the text (line 157)*

**Response:**

Thank you for the comment. In Figure 2, the antibody used to detect H2A ubiquitination specifically targets lysine 119 (K119) of H2A. We used the Ubiquityl-Histone H2A (K119) Rabbit mAb (Cell Signaling, Cat#8240) for this purpose. A detailed description of this antibody and its specificity is now included in the revised manuscript (line 190), and we have also specified the antibody in the Key Resources Table within the supplemental materials for clarity.

*How is the promoter region of SLC7A11 defined (Figure 4)?*

**Response:**

Thank you for your question. In Figure 4, the promoter region of SLC7A11 (Gene ID: 23657) was defined based on the intronic region located upstream of the coding sequence, as annotated in the NCBI database. To amplify this region, we designed gene-specific primers. The sequences of these primer [*SLC7A11*(ChIP-qPCR)] are provided in Table S2 for reference. Their effectiveness in targeting the promoter region was validated through ChIP-PCR (Figure 4E), ensuring accurate amplification of the defined promoter region.

***Reviewer Comments:***

***Reviewer #3***

*Comments:*

*Microsporidia are obligate intracellular fungi that represent a public health problem in immunocompromised humans. We know very little about the mechanisms through which these intracellular parasites manipulate host cell biology. In this study, the authors explore the role of a putative host-modulating protein endospore protein 1 (EnP1) from Encephalitozoon hellem during infection. The authors previously identified the Enp1 as a secreted E. hellem protein that is localized to the host nucleus, where it modulates host ferroptosis sensitivity by interacting with and modifying H2B ubiquitination (Guan et al. 2024). Here the authors turn their focus to another interaction partner they observed, the related histone H2A. In essence the authors do a very thorough job showing that Enp1 seems to also impact the function of H2A; interacting with it and in this case increasing monoubiquinitation of this histone subunit (as opposed to how it decreases the monoubiquinitation of H2B). They show that Enp1 reduces the levels of BAP1 (a ub deubiquitinase that acts on H2A) at the mRNA and protein levels but also interacts with BAP1 physically. BAP1 can clearly antagonize E. hellem infection, H2B levels at the SLC7A11 locus, SLC7A11 expression, and ferroptosis sensitivity. In addition, the authors importantly show that BAP1 overexpression can counteract some of the effects of Enp1 expression on host cells. This is a critical area of research for understanding how microsporidia impact and manipulate host cell biology. The authors are ideally suited to carry out the work based on their previous work and this paper represents a rigorous effort to define mechanistic aspects of host cell modulation by E. hellem specifically via its effector protein EnP1. The manuscript details some very interesting findings and is very convincing. One question is whether this work, which is essentially exploring a second histone target of Enp1 as opposed to a truly novel mechanism of Enp1 in host cell manipulation, is impactful enough to warrant publication in this journal. In addition, there are some questions about the mechanisms at play in regulating H2Aub via BAP1. Finally, the discussion could use some additional points and some reorganization.*

**Response:**

We greatly appreciate the positive and constructive feedback offered by the reviewer on our work. We have diligently worked to address and incorporate the supplementary materials and suggested modifications. In the revised manuscript, we will provide detailed point-by-point responses to the reviewer’s comments.

***Major Comments:***

*1. In Figures 2 and 3, the authors show how various treatments reduces the amount of ubiquitinated H2A. However, the authors do not show total H2A amounts, making it difficult to determine whether there is a reduction in total H2A or only in the amount that is ubiquitinated. In addition to H3 measured as a loading control, the amount of total H2A should be measured and reported.*

**Response:**

We thank the reviewer for this valuable suggestion. To clarify this point, we measured the total cellular levels of H2A under both EnP1 overexpression and microsporidian infection conditions, with H3 included as a loading control. As shown in Figure S2A–S2B, total H2A levels remained unchanged across all conditions. These findings confirm that the reductions observed in Figures 2 and 3 are specific to ubiquitinated H2A (H2Aub) rather than due to changes in the overall expression of total H2A.

Figure S2A-B

*2.The authors provide evidence that EnP1 expression reduces BAP1 mRNA and protein levels. They also show that Enp1 physically interacts with BAP1. As BAP1 overexpression seems to at least partially counteract Enp1 function, it seems that the effects on the BAP1 levels are critical. Mechanistically, the authors should experimentally explore how Enp1 is reducing BAP1 mRNA and protein levels as this is a key mechanistic link that is not elucidated. Similarly, is Enp1 interaction with BAP1 or H2A actually important for the effects they observe? If so, what is Enp1 doing to BAP1?*

**Response:**

We thank the reviewer for raising these important questions. However, there may have been some confusion regarding our findings. As shown in Figure 2O, we actually demonstrated that EnP1 does not directly interact with BAP1, and therefore EnP1 is not a direct regulator of BAP1 expression. Instead, we propose that the increase in H2Aub levels, along with the steric hindrance caused by the interaction between EnP1 and H2A, may play a critical role in modulating BAP1 activity. We hypothesize that the elevated H2Aub levels could contribute to transcriptional repression of key genes, potentially including BAP1, through a negative feedback mechanism. This mechanism might be driven by the global changes in histone modification profiles caused by EnP1 expression. Given the importance of pathogen-derived effector proteins in modulating host gene expression, we agree that this is a crucial area for further investigation, and we are currently exploring this pathway in greater depth.

*3. In the discussion, the authors describe different K sites for ubiquitination. Is it known which site BAP1 usually regulates? If not can they show or comment on which ubiquitination site is primarily affected by Enp1?*

**Response:**

We thank the reviewer for the insightful question. Both RING1B and BAP1, as key modifying enzymes, primarily target lysine 119 (K119) of histone H2A, which is a critical site for the regulation of transcription [8]. Modification at this specific lysine residue is essential for mediating transcriptional regulation. Moreover, H2Aub detected using a modification-specific antibody against H2Aub (K119) was significantly increased in the EnP1 overexpression cell line (Figure 2A and 2B). This result further suggests that lysine 119 (K119) of histone H2A is primarily affected by EnP1.

*Minor Comments:*

*I appreciate that there are few if any examples of microsporidia manipulating host gene expression via nuclear targeted proteins so the discussion of examples from other fungi MoHTR1 and 2 is good. However, maybe some additional examples in fungi would be beneficial.*

*- Inclusion of additional examples of microsporidia manipulation of host cells in other ways would help contextualize the findings here.*

*- Inclusion of additional examples of microsporidia manipulation of host cells.*

**Response:**

We have expanded the Discussion section to include comparisons with effector proteins from other pathogenic fungi, thereby enriching the understanding of effector pleiotropy (as detailed in lines 459-479).

*I did not see the rationale for the inclusion of the statement on Ln344 about how ‘pathogenic proteins can target acidic plaques within host cells’ at all, but especially as the first sentence of this paragraph.*

**Response:**

We have provided an explanation of acidic patches and included examples along with mechanistic descriptions of how pathogens target these structures (lines 483-490).

*I had a hard time following the section of the discussion describing how H2B monoubiquinitation is related to other modifications and the dynamic nature of the SLC7A11 promoter. The authors mention how BAP1 and PRC1 both lead to repression, but then don’t talk about H3K27me3 until the next paragraph. I think introducing the PRC1 to PRC2 link in the paragraph starting on Ln327 or saving the discussion of the link and the dual nature of H2B monoubiquinitation until the next paragraph (Ln386) would make this point clearer.*

**Response:**

We thank the reviewer for their suggestion, which we believe enhances the readability of the manuscript. In response, we have integrated the discussion of H2A monoubiquitination with that of H3K27 methylation, thereby providing a more coherent explanation of both PRC1 and PRC2 and their functional relationship (lines 516-537 and lines 556-565)**.**

*The authors previous work showed that Enp1 impacts increase H2B monoubiquinitation (Guan et al. 2024) which then modulate p53 expression and ultimately alters SLC7A11 levels indirectly. While they allude to this in the final paragraph of the discussion, a more thorough discussion of the interaction of the H2A and H2B regulation by EnP1 in the manipulation of SLC7A11 and ferroptosis is warranted.*

**Response:**

We apologize for any confusion caused. We have now included a comparative explanation of the relationship between H2A and H2B, as well as their respective roles in mediating resistance to ferroptosis in microsporidia (lines 572-579).

*In discussion of Figure 4 in Ln240, I believe H2Aub levels are described instead of the intended SLC7A11.*

**Response:**

We have made the corresponding revisions. Our investigation specifically focuses on the relationship between BAP1 expression levels and SLC7A11(lines 338-339).

**Reference:**

1.	Abramson, J., et al., *Accurate structure prediction of biomolecular interactions with AlphaFold 3.* Nature, 2024. **630**(8016): p. 493-500.

2.	Guan, J., et al., *Microsporidian EnP1 alters host cell H2B monoubiquitination and prevents ferroptosis facilitating microsporidia survival.* Proc Natl Acad Sci U S A, 2024. **121**(34): p. e2400657121.

3.	Li, X., et al., *BRHIS1 suppresses rice innate immunity through binding to monoubiquitinated H2A and H2B variants.* EMBO Rep, 2015. **16**(9): p. 1192-202.

4.	Sun, K., et al., *HapX-mediated H2B deub1 and SreA-mediated H2A.Z deposition coordinate in fungal iron resistance.* Nucleic Acids Res, 2023. **51**(19): p. 10238-10260.

5.	Su, X.M., et al., *ALFIN-like proteins link histone H3K4me3 to H2A ubiquitination and coordinate diverse chromatin modifications in Arabidopsis.* Mol Plant, 2025. **18**(1): p. 130-150.

6.	Barbour, H., et al., *Polycomb group-mediated histone H2A monoubiquitination in epigenome regulation and nuclear processes.* Nat Commun, 2020. **11**(1): p. 5947.

7.	Zhang, Y., et al., *BAP1 links metabolic regulation of ferroptosis to tumour suppression.* Nat Cell Biol, 2018. **20**(10): p. 1181-1192.

8.	Ge, W., et al., *Basis of the H2AK119 specificity of the Polycomb repressive deubiquitinase.* Nature, 2023. **616**(7955): p. 176-182.

9.	Rong, Y., et al., *USP16-mediated histone H2A lysine-119 deubiquitination during oocyte maturation is a prerequisite for zygotic genome activation.* Nucleic Acids Res, 2022. **50**(10): p. 5599-5616.

10.	Gu, Y., et al., *The histone H2A deubiquitinase Usp16 regulates hematopoiesis and hematopoietic stem cell function.* Proc Natl Acad Sci U S A, 2016. **113**(1): p. E51-60.

11.	Jullien, J., et al., *Gene Resistance to Transcriptional Reprogramming following Nuclear Transfer Is Directly Mediated by Multiple Chromatin-Repressive Pathways.* Mol Cell, 2017. **65**(5): p. 873-884.e8.

12.	Koppula, P., L. Zhuang, and B. Gan, *Cystine transporter SLC7A11/xCT in cancer: ferroptosis, nutrient dependency, and cancer therapy.* Protein Cell, 2021. **12**(8): p. 599-620.

13.	Chen, X., et al., *Ferroptosis: machinery and regulation.* Autophagy, 2021. **17**(9): p. 2054-2081.

14.	Li, Q., et al., *Enhancing Ferroptosis-Mediated Radiosensitization via Synergistic Disulfidptosis Induction.* ACS Nano, 2025. **19**(1): p. 1757-1770.

15.	Deng, J., et al., *SPTBN2 suppresses ferroptosis in NSCLC cells by facilitating SLC7A11 membrane trafficking and localization.* Redox Biol, 2024. **70**: p. 103039.

16.	Chen, H., et al., *Patulin disrupts SLC7A11-cystine-cysteine-GSH antioxidant system and promotes renal cell ferroptosis both in vitro and in vivo.* Food Chem Toxicol, 2022. **166**: p. 113255.

17.	Thomas, J.F., et al., *Structural basis of histone H2A lysine 119 deubiquitination by Polycomb repressive deubiquitinase BAP1/ASXL1.* Sci Adv, 2023. **9**(32): p. eadg9832.

18.	Tamburri, S., et al., *Histone H2AK119 Mono-Ubiquitination Is Essential for Polycomb-Mediated Transcriptional Repression.* Mol Cell, 2020. **77**(4): p. 840-856.e5.

19.	Zhang, J., et al., *Disruption of PRC1 components RING1A and RING1B promotes angiogenesis via relieving BMP4 repression.* J Adv Res, 2025.
