# Supplementary material for: EnP1 exploits H2Aub-dependent epigenetic reprogramming to promote microsporidia proliferation in host cells
Source: PLoS Pathog. 2026 Jan 7;22(1):e1013853. doi: 10.1371/journal.ppat.1013853 (PMC12795459; doi:10.1371/journal.ppat.1013853)
Supplement: Attachment — Submitted filename: Response to Reviewers.docx [file ppat.1013853.s008.docx]

The point-by-point answers to the questions, comments, and suggestions are listed below.

***Additional Editor Comments:***

*For all bar graphs in the paper, please plot all the individual datapoints that gave rise to these graphs, together with annotating which of the three independent experimental replicates the datapoints are from. As per Reviewer #2's suggestion please reference SuperPlots (PMID: 32346721, Fig 1D, far right) as an example.*

*Furthermore, the co-IP/WB studies showing interaction between Flag-ENP1 and HA-H2A would benefit from additional negative controls. Specifically, can the authors exclude the possibility that the tags on these constructs are responsible for the interaction? Single-transfected cells do not seem to be an adequate control here: rather, showing a lack of association between e.g. Flag-GFP and HA-H2A, and a lack of association between Flag-ENP and HA-GFP would provide reassurance that the interaction shown is due to ENP1 and H2A themselves, and not their tags*.

**Response:**

We sincerely appreciate the constructive feedback provided by the editors.

In response to the comment regarding the bar graphs, we have now plotted all individual data points that contributed to these graphs and have clearly annotated the data points according to their respective experimental replicates. Data points from the same biological replicate are represented using identical colors and shapes, making it easy to distinguish between replicates. For example, in the case of the independently repeated Western blot analyses, each data point represents the grayscale quantification of one blot (relative to the control group) (see Figure 2B). Similarly, for both absolute and relative qPCR results, we have followed the guidelines outlined in the literature (PMID: 32346721), using distinct colors and shapes to indicate different biological replicates, while technical replicates within each biological replicate are plotted in lighter shades of the same color in the background (see Figures 3B and 3F). We believe this approach significantly enhances the clarity of the data, improves the overall quality of the figures, and provides a more transparent view of the experimental results.

Regarding the co-IP/WB studies, we have taken additional steps to address the concern about the potential influence of the tags on the observed interactions. Specifically, we performed additional negative controls to exclude the possibility that the interaction was due to the tags on the constructs. Our updated experiments show that H2A-HA could not pull-down Flag-GFP, thereby excluding any interaction between the Flag tag and H2A. Similarly, Flag-EnP1 failed to pull down HA-GFP, ruling out any interaction between the HA tag and EnP1. These negative controls help confirm that the observed interaction is between ENP1 and H2A, and not driven by the tags themselves. We have repeated these experiments, and the results consistently support this conclusion. Additionally, we have updated the raw data to include all original graphs and Western blot images. These are now available for review at the following link: https://data.mendeley.com/datasets/bhscx3g5fc/4.

***Reviewer Comments:***

***Reviewer #1***

***Comments:***

*I am satisfied with the significant efforts made by the authors to allay any concerns. The manuscript is ready for publication*.

**Response:**

We are grateful for the reviewer's positive recognition.

***Reviewer Comments:***

***Reviewer #2***

***Comments:***

*Overall, the authors seem to have made an effort to respond to reviewer comments, and I think in several cases this has clarified things or resolved a concern. In other cases, while the authors responses make sense on some level, I still have trouble understanding the results. For example, regarding my comment on Fig 3B (which I mentioned in my review as a specific example, but reflects a question about many of the figures): Given all to potential sources of variation between biological replicates, I find the size of the error bars (very small) and the statistical significance of this difference (p < 0.01) to be surprising. Parasites were from 3 separate batches, so I would expect some error in parasite counts between replicates, and variation in parasite infectivity/viability. I would also expect error/variation in host cell density plating on different days, small differences in host cell growth rates and viability, etc. There is presumably variation in washing away parasites after infection. Then variation in the read-out of parasite numbers at the end of the experiment. As a check to see if my intuition was fooling me, I tried simulating data for 3 replicates that would give similar means and standard deviations to what is depicted in Fig 3B (see attached figure). To get a mean of roughly 1.3x10^7 with a standard deviation of ~0.05 in the control (my estimates based on the author's figure; I assume the error bars represent standard deviations, though I can't find this in the legends), one might measure something like 1.25, 1.30 and 1.35 x10^7 in the 3 different biological replicates. For a technical replicate, I think this would already be pretty high reproducibility; given all the sources of variation I mentioned above, such minimal variation between complex biological replicates would seem to be very difficult to achieve. I'm not sure if there is something else I am missing? I strongly suggest displaying this kind of data using SuperPlots (PMID: 32346721, e.g., Fig 1D, far right), which show all of the individual measurements alongside the mean and standard (and where applicable, color-code the technical replicates within each biological replicate).*

*Because of these sorts of issues, it is unclear to me what to make of the data. And given the relatively small effects in many cases, it is also unclear to me how important the reported differences are parasite replication, and consequently, how impactful this manuscript will be on the field.*

**Response:**

We thank the reviewers for their rigorous scrutiny. To enhance the credibility of the data, we have strictly followed the requirements for visualizing both biological and technical replicates and have indicated specific *P*-values according to the method described in the literature (PMID: 32346721). The data analysis approach has been detailed in the "Statistical Analysis" section of the *Materials and Methods*. As shown in Figures 3B and 3F for the absolute quantitative PCR data, different biological replicates are illustrated with distinct shapes and colors. Similarly, within each biological replicate, the technical replicates are represented by lighter shades of the corresponding color and plotted in the background. To maximize the reliability of the experimental data, we made every effort to maintain consistent passage numbers and states of host cells across different time points and biological replicates, and performed repeated double-blind counts to minimize variations in microsporidian vitality and quantity. All data presented in the manuscript clearly distinguish between biological replicates and their corresponding technical replicates. Thank you for your rigorous and responsible review again.

***Reviewer Comments:***

***Reviewer #3***

*Comments:*

*The authors addressed every weakness/issue/concern I noted in my previous review.*

**Response:**

We extend our thanks for the critical suggestions and affirmative evaluation provided by the reviewer.
